# Transcriptomics of Tasmanian Devil (*Sarcophilus Harrisii*) Ear Tissue Reveals Homogeneous Gene Expression Patterns across a Heterogeneous Landscape

**DOI:** 10.3390/genes10100801

**Published:** 2019-10-12

**Authors:** Alexandra K. Fraik, Corey Quackenbush, Mark J. Margres, Sebastien Comte, David G. Hamilton, Christopher P. Kozakiewicz, Menna Jones, Rodrigo Hamede, Paul A. Hohenlohe, Andrew Storfer, Joanna L. Kelley

**Affiliations:** 1 School of Biological Sciences, Washington State University, Pullman, WA 99164, USA; alexandra.fraik@wsu.edu (A.K.F.); corey.quackenbush@wsu.edu (C.Q.); mark.margres@wsu.edu (M.J.M.); chris.kozakiewicz@wsu.edu (C.P.K.);; 2 Department of Biological Sciences, Clemson University, Clemson, SC 29634, USA; 3 School of Natural Sciences, Hobart, TAS 7001, Australia; sebastien.comte@utas.edu.au (S.C.); d.g.hamilton@utas.edu.au (D.G.H.); menna.jones@utas.edu.au (M.J.); rodrigo.hamedeross@utas.edu.au (R.H.); 4 Vertebrate Pest Research Unit, NSW Department of Primary Industries, 1447 Forest Road, Orange, NSW 2800, Australia; 5 Department of Biological Sciences, University of Idaho, Institute for Bioinformatics and Evolutionary Studies, University of Idaho, 875 Perimeter Drive, Moscow, ID 83844, USA; hohenlohe@uidaho.edu

**Keywords:** RNA-sequencing, conservation genomics, geographic variation, Tasmanian devil, population level, sex-specific expression

## Abstract

In an era of unprecedented global change, exploring patterns of gene expression among wild populations across their geographic range is crucial for characterizing adaptive potential. RNA-sequencing studies have successfully characterized gene expression differences among populations experiencing divergent environmental conditions in a wide variety of taxa. However, few of these studies have identified transcriptomic signatures to multivariate, environmental stimuli among populations in their natural environments. Herein, we aim to identify environmental and sex-driven patterns of gene expression in the Tasmanian devil (*Sarcophilus harrisii*), a critically endangered species that occupies a heterogeneous environment. We performed RNA-sequencing on ear tissue biopsies from adult male and female devils from three populations at the extremes of their geographic range. There were no transcriptome-wide patterns of differential gene expression that would be suggestive of significant, environmentally-driven transcriptomic responses. The general lack of transcriptome-wide variation in gene expression levels across the devil’s geographic range is consistent with previous studies that documented low levels of genetic variation in the species. However, genes previously implicated in local adaptation to abiotic environment in devils were enriched for differentially expressed genes. Additionally, three modules of co-expressed genes were significantly associated with either population of origin or sex.

## 1. Introduction

To understand the genetic basis of the diversity of phenotypes in wild populations inhabiting complex environments, more studies are needed to examine baseline gene expression variation across species’ geographic ranges [1]. Typically, studies of the impact of environmental features on gene expression are conducted in controlled laboratory settings (reviewed in [2,3]). While lab-controlled experiments identify responses to particular stimuli, observed changes in expression may not reflect natural environmental responses [4]. Laboratory experiments may also fail to capture the complexity that multivariate, environmental stimuli exhibit on transcriptomic responses in a natural setting [5]. 

Characterization of gene expression differences in wild populations in situ is particularly relevant for species of conservation concern [6]. Understanding the baseline geographic variation in gene expression differences may help predict species’ adaptive potential in a time of ubiquitous global change. Despite the decreasing costs of sequencing, ecological geneticists studying wild populations are still constrained by a lack of resources including reference genomes and annotations and a priori data regarding putative genes of interest [7,8,9]. In addition, RNA-sequencing (RNA-seq) studies are often limited by a lack of available data regarding the abiotic and biotic environmental variation that might be driving putative changes in gene expression. A combination of long-term mark-recapture studies of the Tasmanian devil, *Sarcophilus harrisii*, across its heterogeneous geographic range [10] and the development of extensive genomic resources [11,12,13,14,15] make the species ideal for studying transcriptional differences among wild populations.

Since 1996, devils have been threatened by a transmissible cancer—devil facial tumor disease (DFTD) [10,15]. Devils appear almost universally susceptible to this fatal disease, which has spread throughout the majority of the species’ geographic range [10]. Low genetic variation in devils [15], owing to historic population bottlenecks [16,17], has been proposed as a key contributor to the rapid spread of DFTD. Additionally, DFTD evades detection via irregular expression of its own major histocompatibility complex (MHC) and the downregulation of devil MHC [18]. As DFTD will soon reach the remaining uninfected populations of devils, it is important to understand the adaptive potential of devils in the face of disease and future environmental change. 

Geographic variation in the extent of population declines [19] and molecular responses to disease [12,13,16,20,21] suggests that there are heritable responses to DFTD that vary among devil populations. The presence of Tasmanian devils across a wide variety of vegetation types [22,23,24], together with spatially explicit genetic variation throughout devil populations [15,16,25,26,27], provide conditions that could promote local adaptation in devils [28]. Using genetic-environmental associations (GEA), we identified 64 genes putatively involved in adaptation to local abiotic environment among seven Tasmanian devil populations [21]. The majority of these genes were strongly associated with climatic variables including mean annual temperature and annual temperature range as well as vegetation cover type, all of which varied significantly among the populations sampled. Following the arrival of DFTD in those populations, the apparent signal of local adaptation to abiotic environment appeared to diminish, likely swamped by the strong selection imposed by the fatal tumor [21]. Understanding the functional role of these adaptive candidates may have important implications for the long-term conservation of Tasmanian devils. One approach to accomplishing this goal is to study baseline transcriptional variation among natural devil populations.

In this study, we investigated how gene expression in the ear tissue of healthy Tasmanian devils varied across the landscape. First, we tested for transcriptome-wide patterns of differential gene expression among three geographically distinct populations while controlling for sex. Second, we tested if candidate genes for adaptation to local abiotic environment were significantly enriched in any of our population comparisons. We hypothesized that significant environmental heterogeneity among Tasmanian devil habitats would generate variable patterns of gene expression. Third, we tested for co-expression and differential expression among sexes while controlling for geographic location. If there is significant sexual dimorphism between male and female devils across populations, then there should be differences in the expression of genes underlying these traits. Finally, we conducted a power analysis to quantify whether we had sufficient power to test for differential expression in our transcriptomic data. 

## 2. Materials and Methods

### 2.1. Sample Collection

**Figure 1:**
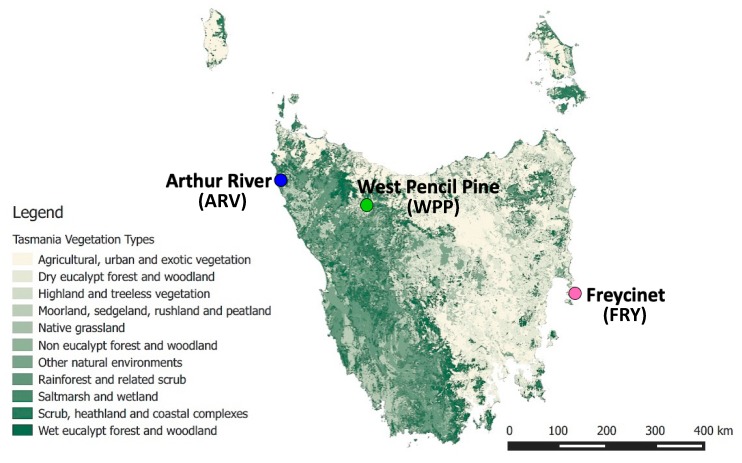
Map of our three geographic sampling locations across Tasmania showing the vegetation distribution across Tasmania.

Phenotypic data and ear tissue biopsies from 20 healthy Tasmanian devils (10 males and 10 females) were collected from May–August 2015 from three different geographic locations across the geographic range of the Tasmanian devil (Figure 1). The populations Freycinet (FRY), Arthur River (ARV) and West Pencil Pine (WPP) were selected as our sampling populations to maximize the geographic and environmental heterogeneity captured in our transcriptomic study (Supplementary Table S1). The population of Freycinet (FRY), the most eastern sampling site, is a rugged granite peninsula primarily dominated by dry eucalypt forest and scrub. It is the driest and warmest of the three sites. West Pencil Pine (WPP) is 211.4 km north-west of FRY, in a wet, subalpine forest including native and non-native eucalypts for commercial harvesting. Away from the sea, it is the highest and coldest study site, with regular frost and snow in winter. Arthur River (ARV), a further 86.4 km north-west of WPP, is a flat coastal site broadly characterized by native grasslands and scrub with patches of coastal forest (Supplementary Table S1).

Regarding ethics approval and consent to participate, animal use was approved under IACUC protocol ASAF#04392 at Washington State University, with approval from the University of Tasmania’s Ethics Committee (A0013326) and a scientific permit from the Tasmanian Department of Primary Industries, Parks, Water and Environment (TFA14228).

Tissue samples were primarily skin, taken from the fleshy epidermis connecting the ear to the rest of the head to reduce discomfort and the likelihood of generating open wounds. All sampled devils were disease-free at the time of sampling in order to avoid capturing transcriptional variation attributable to immune and stress responses to DFTD that might be erroneously attributed to sex or sampling location. Tasmanian devils were trapped using custom-built polyvinyl chloride (PVC) pipe traps of 30 cm in diameter [29]. Forty traps were set over each 25 km^2^ trapping site and baited with meat for ten consecutive trapping nights for the ARV and WPP sites. Due to constraints with land ownership and permissions, FRY was divided into three trapping lines that contained a total of 120 traps over a 160 km^2^ area for seven consecutive nights. Traps were checked after each trapping night, commencing at dawn, and each individual trapped was permanently identified on first capture with a microchip transponder (Allflex NZ Ltd, Palmerstone North, New Zealand). Ear biopsies were taken from the right ear using a Sigma-Aldrich 3 mm biopsy punch and immediately placed into Qiagen RNAlater (RNA*later* RNA stabilizing reagent #76016, Germantown, MD, USAfor storage following the standard sampling protocol from the provider [29]. 

### 2.2. Library Preparation and Sequencing

Each ear tissue sample was placed in a FastPrep-24 (MP Biomedical, Irvine, CA, USA) tube containing 353.5 µL of lysis buffer and ceramic beads. Tissue was then disrupted and homogenized using the MP Biomedical (MP Biomedical Irvine, CA, USA, 116004500) FastPrep-24 tissue lyser for two runs for 40 s at 6.5 m/s each. Total RNA was isolated from lysed tissue using the Nucleospin RNA extraction kit (Macherey-Nagel, Easton, PA, USA, #7420S/L). Extracted RNA yields were quantified using the Qubit 2.0 (Life Technologies, Carlsbad, California, USA) fluorometer using the Qubit RNA High Sensitivity assay kit (Thermo Fisher Scientific, Waltham, MA, USA, #Q32852). mRNA was then isolated from the total RNA using the NEBNext Poly(A) mRNA Magnetic Isolation Module (New England BioLabs, Ipswich, MA, USA, #37490). Directional RNA-seq libraries were produced according to the supplied protocols with cDNA amplification occurring for 14 PCR cycles. Presence of PCR product was visually assessed using an eGel (Thermo Fisher Scientific, Waltham, MA, USA, #G661002). RNA-seq libraries were quantified using a Qubit fluorometer with the HS dsDNA Assay kit (Thermo Fisher Scientific, Waltham, MA, USA, #Q32854). Quality of RNA-seq libraries was assessed on the 2100 BioAnalyzer using the High Sensitivity DNA Analysis kit (Agilent, Santa Clara, CA, USA, #5067-4626). Libraries were then pooled in equal nanomolar concentrations and sequenced across one lane of an Illumina HiSeq 2500 using a 100 base pair (bp) paired-end chemistry at the Washington State University Genomics Core in Spokane, Washington. 

### 2.3. Read Alignment

The HISAT2 v.2.2.1.0 R package was used to align reads to the Tasmanian devil reference genome (Genbank Accession JN216828, [11]) using their best recommended practices for stranded libraries [30]. Following alignment, output SAM files were sorted and converted to BAM files using SAMtools v 1.2 [31]. StringTie v 1.2.4 was used to assemble the alignments into transcripts using the devil reference gene annotation set Ensembl v 7.0.86 [11], producing tables of read counts of only those assembled transcripts which match the reference transcripts. StringTie read count data were then converted into a gene count matrix for gene expression analyses using the provided prepDE.py script (ccb.jhu.edu/software/stringtie/dl/prepDE.py). Using Picard Tools v.1.141 (http://picard.sourceforge.net) *CollectRNASeqMetrics,* we collected summary statistics to evaluate the quality of our transcriptome alignments. In addition to the ear tissue, we mapped the published reads from the Tasmanian devil milk transcriptome [14] to the reference genome to compare mapping rates.

### 2.4. Multidimensional Scaling (MDS)

We used the plotMDS function in the “limma” package in R [32] on the top 1000 genes to determine if either of our covariates of interest (population or sex) explained a significant amount of variation in Euclidean distances in our log-transformed gene expression counts. The top 1000 genes were selected using the “common” option, which selects the same 1000 genes with the largest standard deviations between all individuals compared. We also filtered out all genes that had zero counts and then used pairwise Pearson’s correlates to generate a matrix of Euclidean distance values between samples. In addition to sex and geographic location, we also visually examined whether the date of the ear biopsy collection, RNA extraction, or library preparation described variation in gene expression count data.

### 2.5. Differential Expression Analysis

Variation in gene expression amongst individuals was quantified using the EdgeR [33,34] package in R. Specifically, we tested for differences at the gene level between our variables of interest: Sex and population. Comparisons between populations were pairwise, resulting in three total comparisons for population and one comparison for sex. Genes with no expression in any individual were filtered out. Read counts were normalized for each individual by library size using the calcNormFactors function. Model dispersion was estimated using the estimateDisp function, and generalized linear models (GLMs) were fit to each gene using a quasi-likelihood test function (glmQLFit). We set a false-discovery rate (FDR) threshold of 0.05 to correct for multiple testing and did not set a log-fold change (Log_2_FC) cut-off value. 

The quaslikelihood models (glmQLFit) used in EdgeR to quantify the relationship between gene expression and our covariates of interest (a) sex and (b) sampled populations. 

(a) 0 + Sex + Population

contrast = F, − M

(b) 0 + Population + Sex 

contrast = ARV, − FRY

contrast = WPP, − FRY

contrast = WPP, − ARV

We set the y-intercept to zero and included both variables in our models. We first tested for differential gene expression between the sexes (a). In (b), we tested for differential expression between one population compared to another (i.e., ARV compared to FRY). The contrasts determined which factor was being considered the reference population. For example, contrast = ARV, − FRY described differences in expression between FRY and ARV, in which positive Log_2_FC values would indicate a gene was upregulated in ARV compared to FRY. 

### 2.6. Weighted Gene Co-Expression Network Analysis (WGCNA)

In addition to our gene-specific differential expression analyses, we took a network-based approach to identify clusters (modules) of genes with highly correlated expression patterns. We were ultimately interested in whether modules were associated with population or sex. We first constructed gene expression networks using the “WGCNA” package in R [35] using the gene count matrix produced by StringTie as our input. Only genes expressed in at least one individual were included. Gene counts were converted to log-transformed [log (x + 1)] counts per million (cpm) using the cpm function in EdgeR. These values were used as input for our module detection using step-by-step network construction and consensus module detection. In total, 16,265 genes were included in our WGCNA. We constructed a signed gene network, setting the soft threshold, or power level, to nine, as this was the lowest value that resulted in the optimal topology for which the scale-free topology fit index reached 0.7. Once modules were generated, we tested for associations between each module and our external variables of interest (sex and each of the populations) using Pearson’s correlate. Since we had three populations of interest, we compared the association of modules to a single population relative to the other two (i.e., individuals in ARV vs WPP & FRY), resulting in a total of four tests. Additionally, we tested for the gene ontology (GO) enrichment of modules with significant associations to our populations or sex (FDR < 0.05) using the Gene Ontology Consortium’s online GO enrichment analysis tool [36]. GOs that were functionally enriched were those that were overrepresented in those modules (*p* < 0.05) compared to the background of all genes transcribed in the ear tissue of Tasmanian devils.

### 2.7. Gene Set Enrichment Analysis (GSEA)

To investigate whether any genes previously associated with local adaptation in Tasmanian devils [21] were differentially expressed, we conducted a GSEA. GSEA can be used to evaluate how the changes of expression of groups of genes, or multiple groups of genes, deviate from a reference set of genes [37,38]. In our study, we tested whether the expression of genes putatively involved in adaptation to local abiotic environment were enriched in the ear tissue. To this end, we conducted a GSEAPreranked analysis, creating a list of expressed genes ranked in descending order of Log_2_FC for each pairwise population comparison of interest [37,38]. Log_2_FC values came from the output of EdgeR models for pairwise population differential gene expression analyses. We therefore conducted a total of three separate GSEAPreranked analyses to determine whether candidate genes for local adaptation were overrepresented in the highest or lowest rankings of pairwise differentially expressed genes. 

### 2.8. Power Analysis

We used the R package “RNASeqPower” to determine whether we obtained sufficient power to detect patterns of differential gene expression between our covariates of interest [39]. This program estimates the optimal power and number of replicates per biological condition necessary to detect significant differential gene expression at varying effect sizes. We evaluated whether we had sufficient power to detect both moderately expressed (Log_2_FC = 1.5–2.99) and more highly differentially expressed genes (Log_2_FC ≥ 3) between the biological conditions of sex and geographic location. We used an α = 0.05 and coefficient of variation of 0.72 for sex and 0.74 for populations, which are the values for our expression data as estimated from the biological coefficient of variation function in EdgeR [32]. 

### 2.9. Availability of Data and Materials

The transcriptome data from this article are available at the NCBI Sequence Read Archive (SRA) with accession number PRJNA510591. The code is available at github.com/jokelley/devil-transcriptomics. 

## 3. Results

### 3.1. Sequencing and Alignment

We sequenced RNA from the ear tissue of a total of 20 devils (10 males and 10 females) from three geographic locations (Figure 1, Supplementary Table S2), yielding 23,461,028 ± 5,004,218 reads on average per individual. We retained a total of 454,514,778 reads after trimming and filtering out low-quality reads. An average of 85% of reads mapped to the Tasmanian devil genome [11]. Of these, 56.4% of bases mapped to annotated mRNA regions, 37.4% to intergenic regions, and 6.2% to intronic regions (Supplementary Table S2). The published milk transcriptome [14] aligned to the reference genome at a higher rate than the ear tissue, also with a large number of reads mapping outside of annotated mRNA regions (Supplementary Table S2). 

### 3.2. Multidimensional Scaling Cluster Analysis

**Figure 2:**
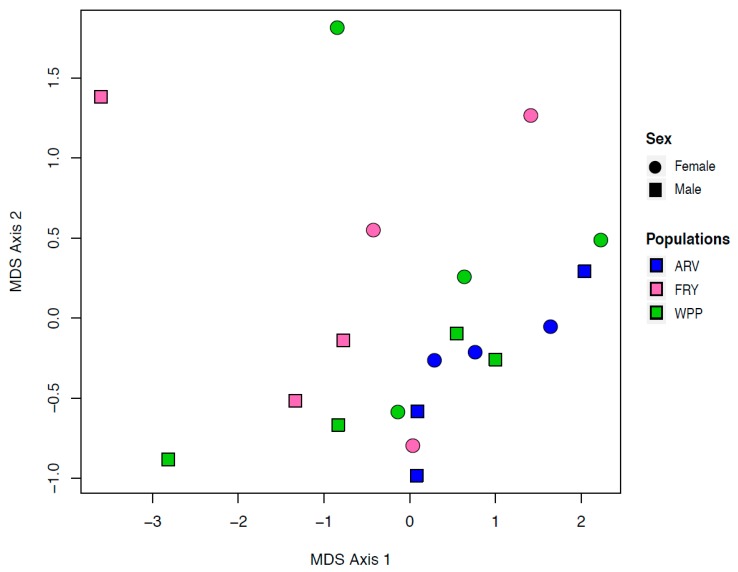
Multi-dimensional scaling (MDS) plot displaying similarity among the samples based on transformed count data. There is no discernible clustering based on sex (circles for females and squares for males) or geographic location.

Of the 20,456 genes in the devil genome annotation set, 16,266 of these genes were expressed in devil ear tissue. There was no discernible pattern in gene expression in our MDS plots that could be attributed to either of our primary traits of interest: Sex and population (Figure 2, Supplementary Figure S1). Additionally, there was no clustering related to the sample collection date, RNA extraction date, or the mRNA library preparation date. 

### 3.3. Identifying Population and Sex-Biased Differential Gene Expression 

**Figure 3:**
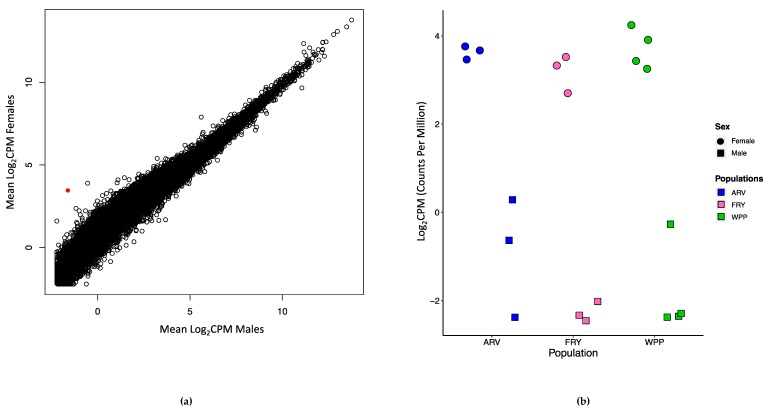
Scatterplot showing the Log_2_CPM (log-transformed counts per million) values between (**a**) males and females, with the red point indicative of the gene *FRMD7*. (**b**) Scatterplot showing the Log_2_CPM values for the gene *FRMD7* in males and females within each population. There was significant differential gene expression in *FRMD7* between the sexes (circles for females and squares for males) across all populations sampled from different geographic locations.

No genes were significantly differentially expressed transcriptome-wide between any pair of sampled populations. Additionally, there was little transcriptional variation between male and female devils (Figure 3a), with only one gene significantly differentially expressed (*FRMD*7, FDR = 0.001, F = 58.18). *FRMD*7 was consistently downregulated in males (Log_2_FC = −5.62) compared to females across all studied populations (Figure 3b). 

### 3.4. Population and Sex-Specific Patterns of Gene Co-Expression 

Three modules of co-expressed genes were significantly associated with sex or population (Supplementary Figure S2). Module28 was significantly associated with both FRY (R^2^ = −0.506, *p* = 0.0228) and ARV (R^2^ = 0.514, *p* = 0.0205) and contained 82 genes (Supplementary Figure S2). Module 28 was enriched for several biological process GO terms including macromolecular metabolism and oxidation-reduction (Supplementary Table S3). Module 3 (48 genes, R^2^ = 0.630, *p* = 0.003) and Module 16 (124 genes, R^2^ = −0.0541, *p* = 0.0138) were significantly associated with sex (Supplementary Figure S2). Module 3 had GO enrichment for structural ribosomal and mitochondrial activity (Supplementary Table S4). Module 16 did not have any GO enrichment.

### 3.5. Gene Set Enrichment Analysis (GSEA)

Of the 64 genes previously identified as candidates for involvement in local adaptation to abiotic environment in Tasmanian devils [21], 59 of these genes of these genes were expressed in the ear (Supplementary Table S4). There were significant variations of the expression of these genes in the WPP vs. FRY (nominal *p*-value = 0.0147, normalized enrichment score = 1.46) pairwise population differential gene expression analyses (Supplementary Figure S3). Eighteen of the 25 genes in the leading edge of the WPP vs FRY GSEA were included in the enrichment, and these genes were broadly associated with GO terms including cellular and external response to stimulus. 

### 3.6. Power Analysis 

Using RNASeqPower, we found we had greater power to detect changes in genes with >3 Log_2_FC differences between sex (90.2%) compared to between populations (74%) (Supplementary Figure S4). For genes that had moderate differential gene expression (1.5–2.99 FC), we had lower power to detect DE between the sexes (22–90%) and populations (18.2–73.99%). 

## 4. Discussion

Significant progress has been made in understanding the genomic and transcriptomic basis of response to severe environmental conditions such as freezing [40,41], high altitude [42,43], and heat shock [44,45]. However, the processes governing variation in gene expression among wild populations occupying less extreme natural landscapes remains relatively unexplored. In this study, we examined patterns of gene expression variation in ear tissue among populations of the Tasmanian devil. Previous genetic studies have found low levels of standing genetic variation in devil populations, with evidence of some genetic structure across the heterogeneous landscape of Tasmania [15,25,26]. We hypothesized that significant environmental heterogeneity among the sampled populations might generate selective pressures that would lead to variation in gene expression. However, we found no genes significantly differentially expressed among our sampled devil populations, and only one gene (*FRMD7*) was significantly differentially expressed between the sexes. Although there was no significant transcriptome-wide variation in differential gene expression across the landscape, we found modules of co-expressed genes significantly associated with ARV and WPP. Additionally, we found an enrichment of putative locally adapted genes when comparing expression between FRY and WPP. 

### 4.1. Devil Ear Tissue Lacks Transcriptome-Wide Differential Gene Expression

The low levels of differential gene expression transcriptome-wide between sampling locations in the ear tissue, despite demonstrable variation in abiotic environment (Supplementary Table S1), do not necessarily indicate that these populations are not adapted to their local environments. It is possible that the lack of genetic variation among Tasmanian devil populations results in low levels of transcriptional variation along these environmental gradients. However, the lack of differential expression could also reflect a low power to detect small changes in gene expression in response to multiple, concurrent environmental stressors. Indeed, while there was sufficient power to detect large changes in differential expression (Log_2_FC > 3) between geographic locations, we had lower power to detect moderate, more subtle patterns of differential gene expression (Log_2_FC = 1.5–2.99). Other studies appeared to suffer similarly from a low power to detect minorly differentially expressed genes between biological or experimental conditions [3]. However, differentially expressed genes with small fold changes may be important in regulating the response to subtle environmental variation. In *Drosophila melanogaster*, identical individuals reared in control replicate labs experienced microenvironmental variation that stimulated small, but significant changes in differential gene expression [46]. To improve the detection of these changes, larger sample sizes may be necessary to increase power to detect moderate gene expression changes in response to environmental variation across the landscape. 

### 4.2. Differential Expression of Candidate Genes for Local Adaptation 

The differential gene expressions of previously identified candidate genes for local adaptation were found to be significantly enriched in the WPP vs. FRY population pairwise comparison (Supplementary Figure S4). The other two pairwise population comparisons did not have significant enrichment, possibly reflecting the difference in sampling locations between the studies [21]. The mean annual temperature and annual temperature range and seasonal precipitation differ between WPP and FRY, possibly necessitating localized plastic and/or adaptive physiological responses from devils (Supplementary Table S1). These three abiotic variables were found to be significantly correlated with allele frequencies of candidate genes [21]. For example, candidate gene *GBA3* was downregulated in FRY relative to WPP (Log_2_FC = −2.819). Similar downregulation has been observed in sea cucumbers entering aestivation at high water temperatures [47]. The enrichment of candidate genes for local adaptation suggests that environmental variation may play a role in driving patterns of differential expression between these two sampling sites. 

### 4.3. Gene Co-Expression Patterns Reflect Possible Adaptive Responses to Coastal Environments

A module of co-expressed genes (Module 28) was independently, significantly correlated with the two coastal populations. One gene included in Module 28 and previously identified as a candidate for local adaptation in Tasmanian devils was *NPR1* [21]. *NPR1* has been found to be putatively involved in kidney and brain sodium excretion responses in mice and ducks [48,49] and was strongly associated with abiotic environmental variation in three coastal populations (FRY, Woolnorth and Forestier) in a previous landscape genomics study [21]. Other genes in this module have been implicated in living in variable salinity environments, including *FOXA1* [50], *MGST1* [51], and *TSPAN13* [52]. These findings may reflect a greater role for salinity as an environmental pressure in coastal environments than previously appreciated. For example, in coastal areas such as FRY and ARV, devils frequently use the beach and coastal heathland for foraging [53]. Differences in the diets of coastal populations, compared to inland populations, also include the consumption of marine life including seabirds, seals, fish and whales [54].

### 4.4. Sex-Biased Expression Patterns

Sex bias in the expression of *FRMD7*, which was upregulated in female relative to male devils, has also been observed in sheep [55]. *FRMD7* is primarily associated with eye movement, eye control, and neurite development [56,57], as well as a number of X-linked genetic disorders [58,59]. In addition to its role in eye development and movement, a subdomain of *FRMD7* shares structural similarity to Acyl–CoA-binding proteins, which has interesting implications for epidermal functions [60]. The Acyl–CoA-binding protein is involved in fatty acid metabolism, which is crucial for hair and skin maintenance [61]. The interference of the expression of this protein has been shown to cause severe skin and hair abnormalities, even resulting in alopecia and scaling of skin [59]. Sex-based differences have also been observed in regard to DFTD, with females suffering less decline in overall body condition when infected [62] and having different SNPs associated with survival compared to males [12]. Taken together, this evidence suggests a more in-depth investigation of sex-biased molecular variation in devil populations is warranted. 

### 4.5. Choice of Tissue

The lack of significant variation in gene expression across environments could be partially explained by our choice of tissue. Biopsies taken from the skin on the ear have been the only sample taken consistently from both infected and uninfected devil populations for the past 19 years. While some studies have characterized sex-specific patterns of differential expression among tissue types in mammals [63,64,65], insects [66], and fish [67,68], few have focused on expression differences in the skin. Relative to other tissues, human skin had fewer differentially expressed genes but a substantial number of co-expressed genes between males and females [63,69]. While sex-biased patterns of expression in skin exist, they may be subtle, possibly encompassing many genes of small effect, making this variation more challenging to detect. 

We found that increasing the sample size may yield appropriate power to detect low to moderate differential gene expression. Our study could be improved by a higher-quality reference genome and annotation. An unexpected number of transcripts in our reference-based alignments of the ear transcriptomes mapped to intergenic regions rather than mRNA. Our realignment of the published milk transcriptome [14] had a greater than expected percentage of reads mapping to intergenic regions, but at a lower rate than our alignments (Supplementary Table S2). The high number of reads mapping to intergenic regions across studies suggests that a new reference genome and annotation may be necessary to improve the resolution of future genomic and transcriptomic studies of Tasmanian devils. 

## 5. Conclusions

Lack of transcriptome-wide variation in expression in ear tissue of Tasmanian devils among geographically disparate populations highlights the challenge of disentangling the nuances of gene expression patterns in natural settings. This study suggests that tissue selection may be an important, limiting factor in studies seeking to characterize baseline gene expression among multiple populations. Future transcriptomic studies of Tasmanian devils should utilize multiple, different tissue samples to determine if there is variation in expression among tissue types. Additionally, this study demonstrated the challenge of detecting gene expression changes between populations, even those occupying heterogeneous environments. We detected enrichment of differentially expressed candidate genes in two of our populations, suggesting that *a priori* data or a candidate-based approach may improve detection of responses to multiple, concurrent environmental factors. 

## Supplementary Materials

The following are available online at https://www.mdpi.com/2073-4425/10/10/801/s1, Figure S1: Sample dendogram and trait heatmap, Figure S2: Weighted gene co-expression module heat map, Figure S3: Differential gene expression of landscape genomic candidate genes, Figure S4: Power analysis between sex and populations, Table S1: Abiotic environmental centroid values among populations, Table S2: Alignment rates from devil ear tissue and the published devil milk transcriptome, Table S3: Gene-ontology enrichment analysis of co-expressed genes associated with FRY and ARV, Table S4: Gene-ontology enrichment analysis of co-expressed genes associated with sex.
