# Supplementary material for: Transcriptomics of Tasmanian Devil (Sarcophilus Harrisii) Ear Tissue Reveals Homogeneous Gene Expression Patterns across a Heterogeneous Landscape
Source: Genes (Basel). 2019 Oct 12;10(10):801. doi: 10.3390/genes10100801 (PMC6826840; doi:10.3390/genes10100801)
Supplement: Supplementary file 1 [file genes-10-00801-s001.pdf]

Supplementary materials for:

Tasmanian devil (*Sarcophilus harrisii*) populations lack differential gene expression between sex and geographic location in ear tissue

Alexandra K. Fraik[1], Corey Quackenbush[1], Mark J. Margres[1,2], Sebastien Comte[3,4], David G. Hamilton[3], Christopher Kozakiewicz[1], Menna Jones[3], Rodrigo Hamede[3], Paul A. Hohenlohe[5], Andrew Storfer[1,*], Joanna L. Kelley[1]

Affiliations:

[1]*School of Biological Sciences, Washington State University Pullman, WA 99164, USA*

[2]*Department of Biological Sciences, Clemson University, Clemson, SC 29634*

[3]*School of Natural Sciences, Hobart, TAS 7001, Australia*

[4]*Vertebrate Pest Research Unit, NSW Department of Primary Industries, 1447 Forest Road, Orange, New South Wales 2800, Australia*

[5]*Department of Biological Sciences, University of Idaho, Institute for Bioinformatics and Evolutionary Studies, University of Idaho, 875 Perimeter Drive, Moscow, Idaho 83844,*

	Mean Annual Temperature (˚C)	Precipitation Seasonality (mm)	Isothermality	Annual Temperature Range (˚C)	Enhanced Vegetation Index (EVI)	Length of Sealed Roads (km)	Elevation (m)	Surface Area of Water (m^2)
**Arthur River (ARV)**	12.59	34.01	49.73	12.30	52.46	9.96	48.00	280917.49
**Freycinet (FRY)**	12.67	14.51	54.09	17.24	74.01	61.65	57.17	4814676.77
**West Pencil Pine (WPP)**	7.98	31.17	47.98	17.11	61.24	245.23	703.74	77396.95

Supplementary Table 1: Abiotic environmental centroid values among populations. The centroid values for the eight abiotic environmental variables and the biotic variable utilized in the GEA associations for each population sampled. We plotted the location of every sampled Tasmanian devil and calculated the centroid for each sampling area. We then constructed a circular buffer with a 5 km radius around each centroid to represent the sampled area for each study site, which we then used to extract the environmental data. These environmental data were obtained from www.ga.gov.au, www.worldclim.org, and www.thelist.tas.gov.au (see Supplementary Table 1 for a list of the eight abiotic environmental variables we included as qualitative descriptors of the sampling locations). We used ArcGIS version 10 (ESRI 2011) for all spatial analyses.

Sample ID	Population	Sex	Number Reads Pre-Trimming	Number Reads Post-Trimming	HiSat2 Mapping Percent	Percent Coding Bases	Percent UTR Bases	Percent Intronic Bases	Percent Intergenic Bases	Percent mRNA Bases
**1**	ARV	Female	21,087,038	19,833,002	83.7	51.0	8.7	5.9	34.4	59.7
**2**	ARV	Male	21,508,474	20,618,302	85.6	49.4	9.1	4.9	36.6	58.5
**3**	ARV	Male	24,409,004	23,803,520	90.0	42.8	9.3	9	38.9	52.1
**4**	ARV	Female	30,892,554	30,086,866	89.2	46.9	9.6	5.9	37.6	56.5
**5**	ARV	Male	21,059,740	19,895,594	83.7	47.8	9.2	5.5	37.4	57.1
**6**	ARV	Female	30,273,938	29,467,814	89.1	46.4	9.6	5.9	38.1	56.0
**7**	FRY	Female	20,457,152	20,053,534	82.7	47.3	9.5	6.0	37.2	56.8
**8**	FRY	Male	20,389,108	19,612,834	81.7	47.0	10.0	5.5	37.6	57.0
**9**	FRY	Female	28,636,486	27,858,674	87.9	49.0	9.7	5.1	36.2	58.7
**10**	FRY	Female	19,282,898	18,748,094	88.4	47.1	8.8	8.7	35.5	55.9
**11**	FRY	Male	22,524,552	22,000,826	81.6	47.0	9.1	6.7	37.1	56.2
**12**	FRY	Male	13,126,976	12,821,570	78.9	47.1	10.0	5.5	37.4	57.2
**13**	WPP	Female	20,175,034	19,354,872	82.2	50.3	9.0	5.8	34.8	59.4
**14**	WPP	Male	20,659,934	20,012,910	82.4	48.5	9.3	5.7	36.5	57.8
**15**	WPP	Female	28,023,276	27,318,236	87.5	47.9	9.7	5.9	36.4	57.7
**16**	WPP	Male	30,090,240	29,205,678	88.9	46.9	9.5	6.3	37.4	56.3
**17**	WPP	Female	33,284,436	32,391,218	91.8	43.8	8.9	8.7	38.6	52.7
**18**	WPP	Female	20,433,336	19,910,918	87.1	47.8	9.8	6.3	36.2	57.6
**19**	WPP	Male	18,643,388	18,092,414	79.8	41.9	10.4	6.4	41.3	52.3
**20**	WPP	Male	24,100,656	23,427,902	85.0	47.7	9.2	5.9	37.2	56.9
**Ear Sample Averages**			23,461,028	22,733,820	85.0	46.8	9.5	6.2	37.4	56.3
**Milk Transcriptome**	Zoo	Female	2,435,1459,062	22,838,662,422	93.8	66.9	5.8	4.5	22.8	72.7

Supplementary Table 2: Alignment rates from devil ear tissue and the published devil milk transcriptome. HISAT2 alignment rates and mapping statistics for the transcriptomes produced from the ear tissue as well as the published milk transcriptome downloaded from NCBI (accession #PRJNA510591). The three sampled populations included Arthur River (ARV), West Pencil Pine (WPP) and Freycinet (FRY).

GO Term	Genes	FDR
Biological Processes		
Macromolecule metabolic process (GO:0043170)	6	0.003
Cellular macromolecule metabolic process (GO:0044260)	5	0.034
Oxidation-reduction process (GO:0055114)	13	0.038
Lipid metabolic process (GO:0006629)	13	0.046
Molecular Function		
Binding (GO:0005488)	36	0.000
Protein binding (GO:0005515)	24	0.000
Cellular Component		
Cell part (GO:0044464)	48	0.004
Intracellular (GO:0005622)	40	0.007
Membrane-bounded organelle (GO:0043227)	31	0.008
Organelle part (GO:0044422)	19	0.002
Nucleus (GO:0005634)	13	0.023
Cytosol (GO:0005829)	8	0.018
Organelle lumen (GO:0043233)	8	0.018
Intracellular organelle lumen (GO:0070013)	8	0.016
Membrane-enclosed lumen (GO:0031974)	8	0.015

Supplementary Table 3: **Gene-ontology enrichment analysis of co-expressed genes associated with FRY and ARV.** Gene-ontology enrichment analysis results for co-expressed genes in Module28 which was strongly associated with the FRY and ARV populations.

GO Term	Genes	FDR
Molecular Function		
Structural constituent of ribosome (GO:0003735)	7	0.001
Structural molecule activity (GO:0005198)	7	0.043
Cellular Component		
Ragulator complex (GO:0071986)	2	0.057
RNA polymerase I complex (GO:0005736)	2	0.049
Ribosome (GO:0005840)	7	0.000
Ribosomal subunit (GO:0044391)	4	0.047
Mitochondrial protein complex (GO:0098798)	5	0.034
Mitochondrial part (GO:0044429)	7	0.045
Ribonucleoprotein complex (GO:1990904)	8	0.034
Mitochondrion (GO:0005739)	13	0.001
Cytoplasmic part (GO:0044444)	28	0.003

Supplementary Table 4: Gene-ontology enrichment analysis of co-expressed genes associated with sex. Gene-ontology enrichment analysis results for co-expressed genes in Module3 which was strongly associated with sex.

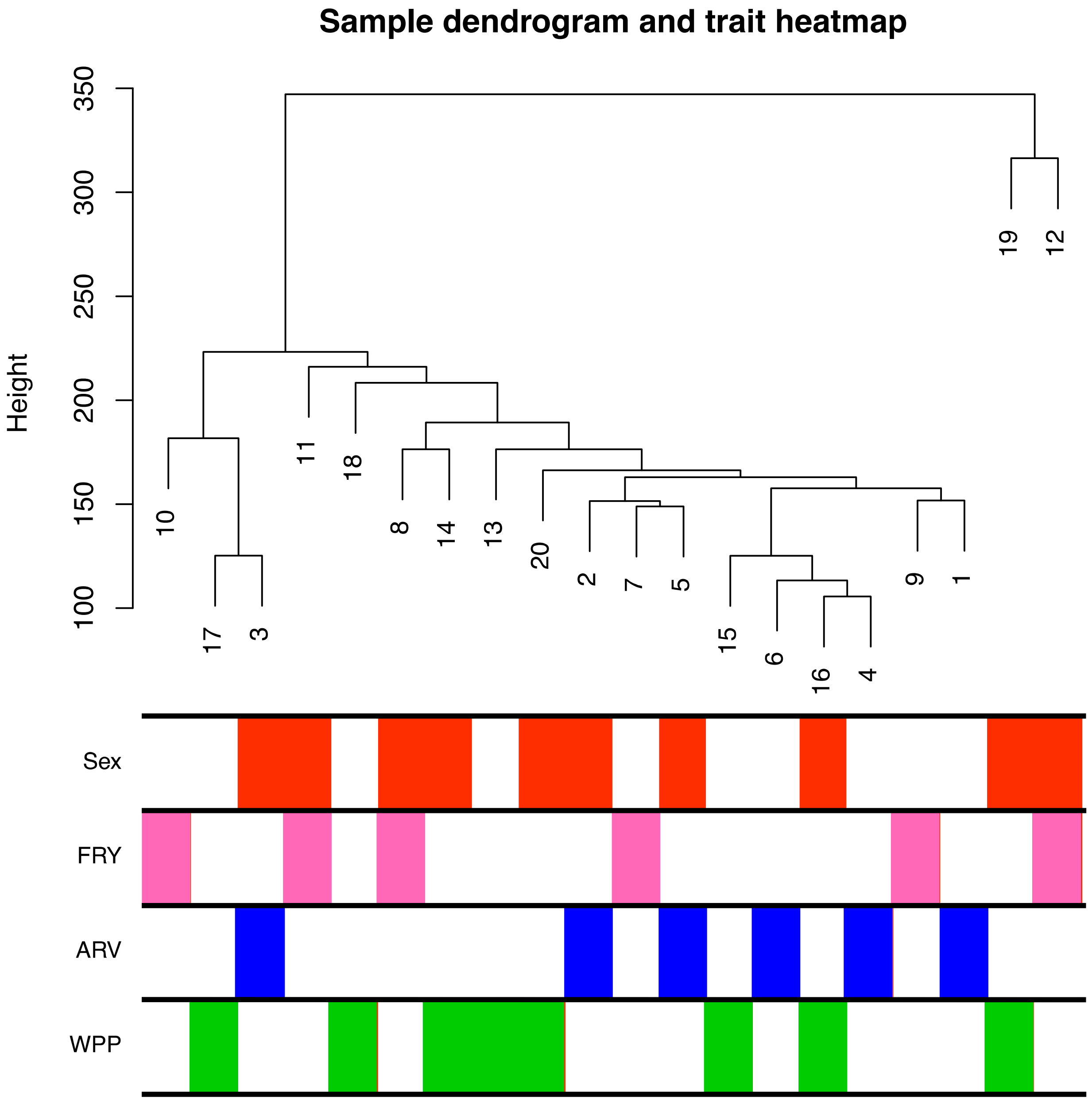

Supplementary Figure 1: Sample dendogram and trait heatmap. Dendrograms of samples showing clustering patterns based on population and sex. Colored blocks indicate assignment of a specific sample (numbered 1-20) to the variables of interest: geographic location or sex.

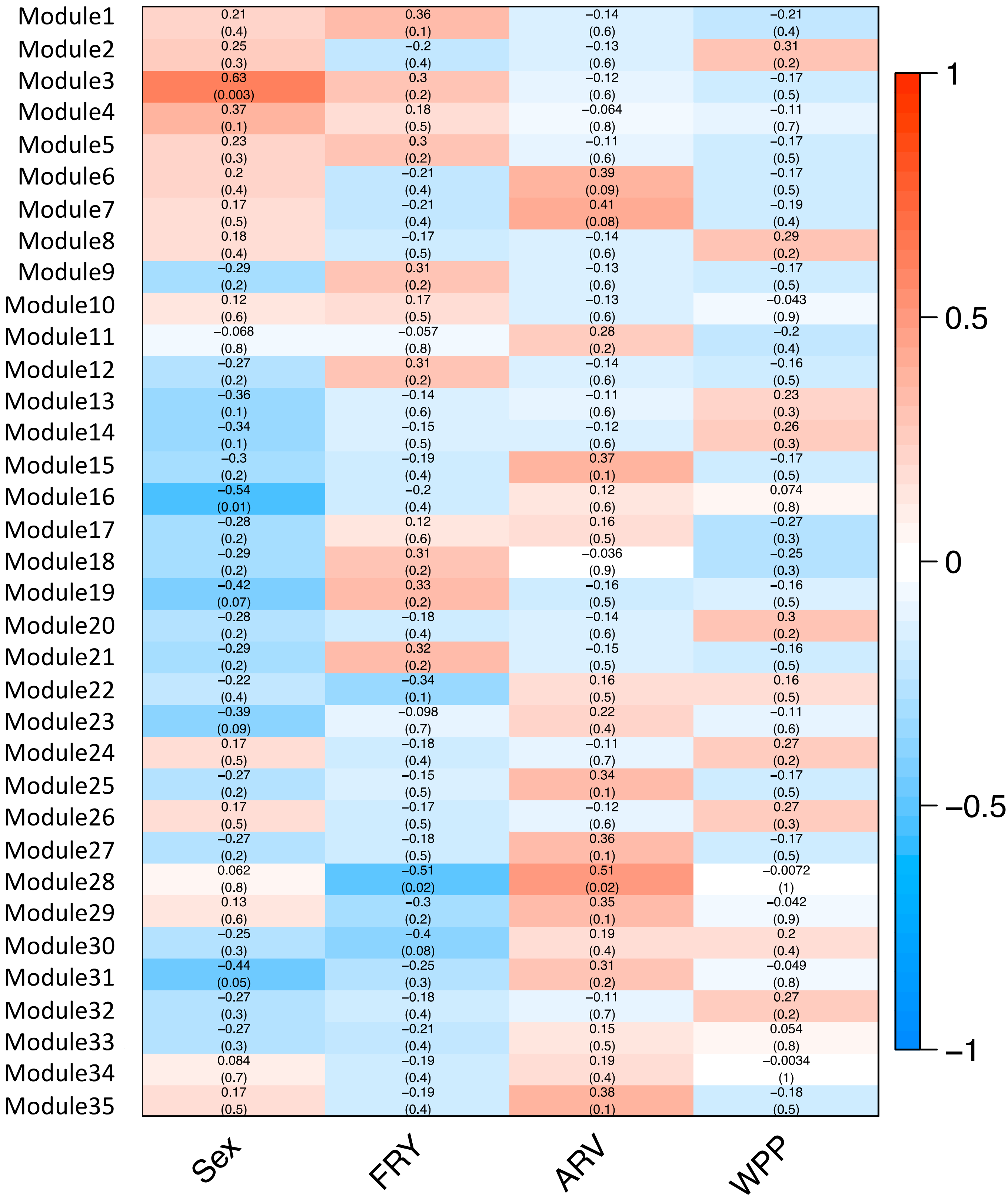
Module1 0.21 (0.4) 0.36 (0.1) −0.14 (0.6) −0.21 (0.4)
Module2 0.25 (0.3) −0.2 (0.4) −0.13 (0.6) 0.31 (0.2)
Module3 0.63 (0.003) 0.3 (0.2) −0.12 (0.6) −0.17 (0.5)
Module4 0.37 (0.1) 0.18 (0.5) −0.064 (0.8) −0.11 (0.7)
Module5 0.23 (0.3) 0.3 (0.2) −0.11 (0.6) −0.17 (0.5)
Module6 0.2 (0.4) −0.21 (0.4) 0.39 (0.09) −0.17 (0.5)
Module7 0.17 (0.5) −0.21 (0.4) 0.41 (0.08) −0.19 (0.4)
Module8 0.18 (0.4) −0.17 (0.5) −0.14 (0.6) 0.29 (0.2)
Module9 −0.29 (0.2) 0.31 (0.2) −0.13 (0.6) −0.17 (0.5)
Module10 0.12 (0.6) 0.17 (0.5) −0.13 (0.6) −0.043 (0.9)
Module11 −0.068 (0.8) −0.057 (0.8) 0.28 (0.2) −0.2 (0.4)
Module12 −0.27 (0.2) 0.31 (0.2) −0.14 (0.6) −0.16 (0.5)
Module13 −0.36 (0.1) −0.14 (0.6) −0.11 (0.6) 0.23 (0.3)
Module14 −0.34 (0.1) −0.15 (0.5) −0.12 (0.6) 0.26 (0.3)
Module15 −0.3 (0.2) −0.19 (0.4) 0.37 (0.1) −0.17 (0.5)
Module16 −0.54 (0.01) −0.2 (0.4) 0.12 (0.6) 0.074 (0.8)
Module17 −0.28 (0.2) 0.12 (0.6) 0.16 (0.5) −0.27 (0.3)
Module18 −0.29 (0.2) 0.31 (0.2) −0.036 (0.9) −0.25 (0.3)
Module19 −0.42 (0.07) 0.33 (0.2) −0.16 (0.5) −0.16 (0.5)
Module20 −0.28 (0.2) −0.18 (0.4) −0.14 (0.6) 0.3 (0.2)
Module21 −0.29 (0.2) 0.32 (0.2) −0.15 (0.5) −0.16 (0.5)
Module22 −0.22 (0.4) −0.34 (0.1) 0.16 (0.5) 0.16 (0.5)
Module23 −0.39 (0.09) −0.098 (0.7) 0.22 (0.4) −0.11 (0.6)
Module24 0.17 (0.5) −0.18 (0.4) −0.11 (0.7) 0.27 (0.2)
Module25 −0.27 (0.2) −0.15 (0.5) 0.34 (0.1) −0.17 (0.5)
Module26 0.17 (0.5) −0.17 (0.5) −0.12 (0.6) 0.27 (0.3)
Module27 −0.27 (0.2) −0.18 (0.5) 0.36 (0.1) −0.17 (0.5)
Module28 0.062 (0.8) −0.51 (0.02) 0.51 (0.02) −0.0072 (1)
Module29 0.13 (0.6) −0.3 (0.2) 0.35 (0.1) −0.042 (0.9)
Module30 −0.25 (0.3) −0.4 (0.08) 0.19 (0.4) 0.2 (0.4)
Module31 −0.44 (0.05) −0.25 (0.3) 0.31 (0.2) −0.049 (0.8)
Module32 −0.27 (0.3) −0.18 (0.4) −0.11 (0.7) 0.27 (0.2)
Module33 −0.27 (0.3) −0.21 (0.4) 0.15 (0.5) 0.054 (0.8)
Module34 0.084 (0.7) −0.19 (0.4) 0.19 (0.4) −0.0034 (1)
Module35 0.17 (0.5) −0.19 (0.4) 0.38 (0.1) −0.18 (0.5)
Sex
FRY
ARV
WPP
1
0.5
0
−0.5
−1

Supplementary Figure 2: Weighted gene co-expression module heat map. Weighted gene co-expression module heat map with gene modules of co-expressed genes occupying the rows and the covariates of interest representing the column values. The first column represents the comparison between modules of gene co-expression and sex. Each subsequent column value represents a population pairwise comparison (i.e., ARV is the comparison of the Arthur River population to the combination of the Freycinet and West Pencil Pine populations). The values in the block above the value in the parentheses are Pearson's correlate describing the strength of the relationship between each gene module and each covariate of interest. Values in the parentheses are the p-values indicating the significance of the relationship between gene module and covariate of interest. Cold colors indicate a negative relationship between the Pearson's correlate between the module and covariate while warm colors indicate a positive relationship.

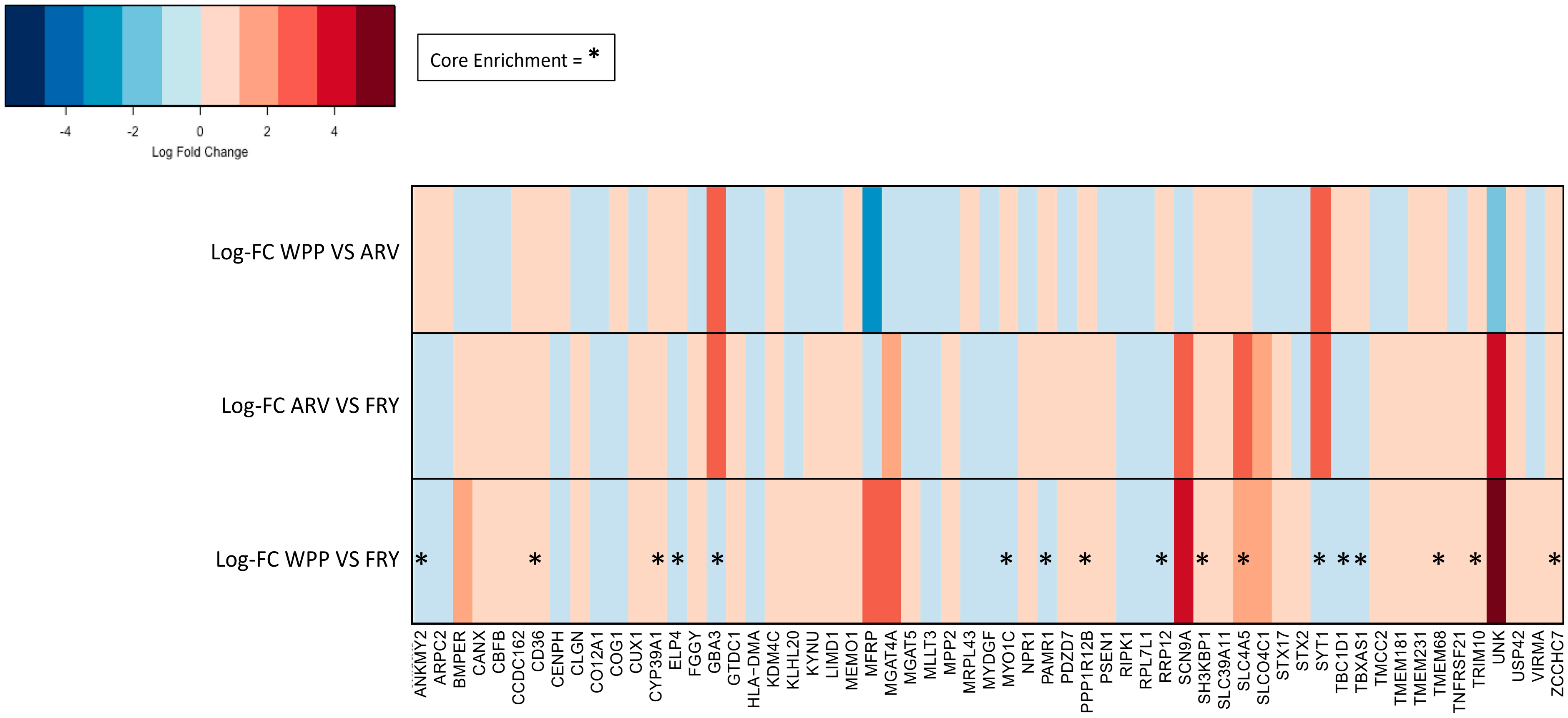

-4
-2
0
2
4
Log Fold Change
Core Enrichment = *
Log-FC WPP VS ARV
Log-FC ARV VS FRY
Log-FC WPP VS FRY
ANKMY2
ARPC2
BMPER
CANX
CBFB
CCDC162
CD36
CENPH
CLGN
CO12A1
COG1
CUX1
CYP39A1
ELP4
FGGY
GBA3
GTDC1
HLA-DMA
KDM4C
KLHL20
KYNU
LIMD1
MEMO1
MFRP
MGAT4A
MGAT5
MLLT3
MPP2
MRPL43
MYDGF
MYO1C
NPR1
PAMR1
PDZD7
PPP1R12B
PSEN1
RIPK1
RPL7L1
RRP12
SCN9A
SH3KBP1
SLC39A11
SLC4A5
SLCO4C1
STX17
STX2
SYT1
TBC1D1
TBXAS1
TMCC2
TMEM181
TMEM231
TMEM68
TNFRSF21
TRIM10
UNK
USP42
VIRMA
ZCCHC7

Supplementary Figure 3: Differential gene expression of landscape genomic candidate genes. Heatmap showing the Log_2FC (Log-transformed fold change) for each of the candidate genes for local adaptation to abiotic environment in a Tasmanian devils landscape genomics (LG) study identified by Fraik et al. 2019. Asterisks indicate these genes were significantly enriched in the leading edge of significant gene set enrichment analyses. Only pairwise population comparisons in gene expression between FRY & ARV and WPP & FRY were significant.

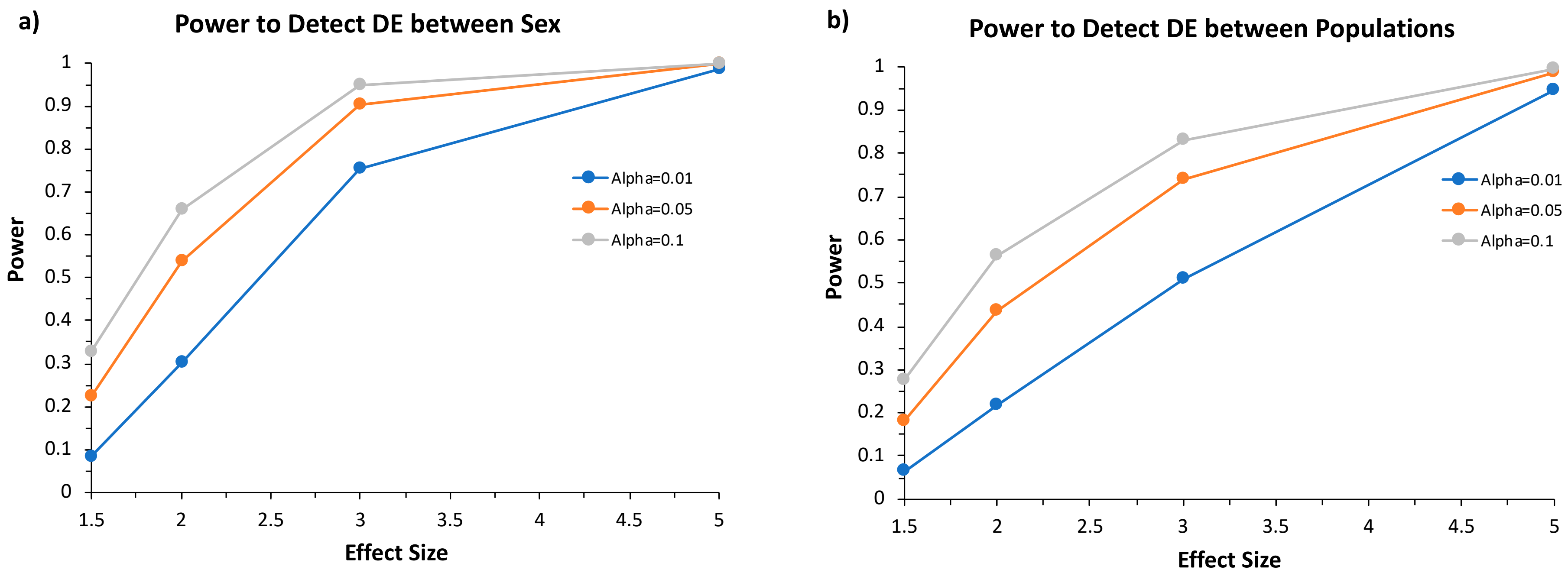

Supplementary Figure 4: Power analysis between sex and populations. Power analysis was conducted to quantify whether we had sufficient power to identify significant variation in expression of genes between our biological conditions. Our experimental design had significant power (> 70%) to detect differential gene expression at an α=0.05 between the sexes (a) and geographic sampling locations (b) at higher effect sizes ($Log_2FC > 3$).
